# The Oil Glands

**Published:** 1888-11

**Authors:** 


### THE OIL GLANDS.

We clip the following very instructive article from a late number of the *Youth's Companion*:

Nearly two and a half millions of sweat glands pour out upon the surface of the body a watery fluid, which aids in keeping the skin soft, and, by its evaporation, in regulating the bodily temperature.

Besides these sweat glands the skin contains the so-called sebaceous glands, that exude an opaque and oily matter. The ducts convey it either directly to the surface or into the upper portion of the hair follicles —the cavities from which the hair proceeds.

The oil is designed to help keep the skin supple, and especially to promote the softness of the hair. These glands are absent from the under part of the feet and hands, and are most abundant in the scalp, face, canal of the ear, and about the nose and mouth. Those in the ear secrete the ear wax. An excess in the secretion renders the face shiny; a deficiency renders the skin and hair dry and harsh.

The glands are sometimes obstructed when the oil becomes thick, or when there is a neglect of cleanliness. This gives rise to *papulæ*, or pimples, which, on being pressed out through the narrow mouth of the oil duct, resemble worms, or grubs. They are frequently called worms, naturally enough, since the hardened dirt on the outer end looks like a head; but they are only thickened oil, though occasionally a minute living worm chooses one of them for his abode.

Occasionally the more fluid elements of the oil are absorbed, leaving only the solid, and these harden into cutaneous calculi. Or the obstructed secretions—yellow, half liquid and half solid, like putty or mortar—may form small tumors on the eyebrows, face, neck and head. They should be cut out when small, or destroyed with caustic. Still, their only harm is in the deformity they cause.

The various forms of acne, or pimple, are due to inflammations of these glands. They occur mainly at the period of puberty and in the years immediately following, and are regarded as due to the constitutional changes then in process. There is at that period a languid and torpid condition of the skin, a tendency to the accumulation of sebaceous matter, and a congestion of the coats of the follicles.

The treatment consists in removing any exciting cause that may exist, improving the nutritive power of the skin and the general system, and stimulating the parts affected. There should also be close attention to diet and habits of life.

